# Supplementary material for: Continuous scanning full-field OCT for fast volumetric imaging of multi-cellular aggregates
Source: Biomed Opt Express. 2026 Feb 4;17(3):1163–72. doi: 10.1364/BOE.578177 (PMC13064596; doi:10.1364/BOE.578177)
Supplement: Supplement 1 [file boe-17-3-1163-s001.pdf]

Supplemental Document

Biomedical Optics EXPRESS

Continuous scanning full-field OCT for fast volumetric imaging of multi-cellular aggregates: supplement

C. KERSUZAN,[1,2,*] A. JANA,[1,2,3] AND A. BADON[1,2]

[1] *LP2N, Laboratoire Photonique Numérique et Nanosciences, Univ. Bordeaux, F-33400 Talence, France*
[2] *Institut d Optique Graduate School & CNRS UMR 5298, F-33400 Talence, France*
[3] *Treefrog Therapeutics, Pessac F-33600, France*
[*] *charlie.kersuzan@u-bordeaux.fr*

This supplement published with Optica Publishing Group on 4 February 2026 by The Authors under the terms of the Creative Commons Attribution 4.0 License in the format provided by the authors and unedited. Further distribution of this work must maintain attribution to the author(s) and the published article's title, journal citation, and DOI.

Supplement DOI: https://doi.org/10.6084/m9.figshare.30616007

Parent Article DOI: https://doi.org/10.1364/BOE.578177

Continuous scanning full-field OCT for fast volumetric imaging of multi-cellular aggregates : supplemental document

C. KERSUZAN,[1,2,*] A. JANA,[1,2,3] AND A. BADON[1,2]

[1]LP2N, Laboratoire Photonique Numérique et Nanosciences, Univ. Bordeaux, F-33400 Talence, France
[2]Institut d Optique Graduate School & CNRS UMR 5298, F-33400 Talence, France
[3]Treefrog Therapeutics, Pessac F-33600, France
[*]charlie.kersuzan@u-bordeaux.fr

This document provides supplementary information to "Continuous scanning full-field OCT for fast volumetric imaging of multi-cellular aggregates". It provides details related to the effect of various parameters on the performance of the FF-OCT approach and theoretical details to demonstrate the validity of the continuous scan approach.

1. Effect of different parameters on continuous scanning FF-OCT images

1.1. Different speed, constant number of images per PSD

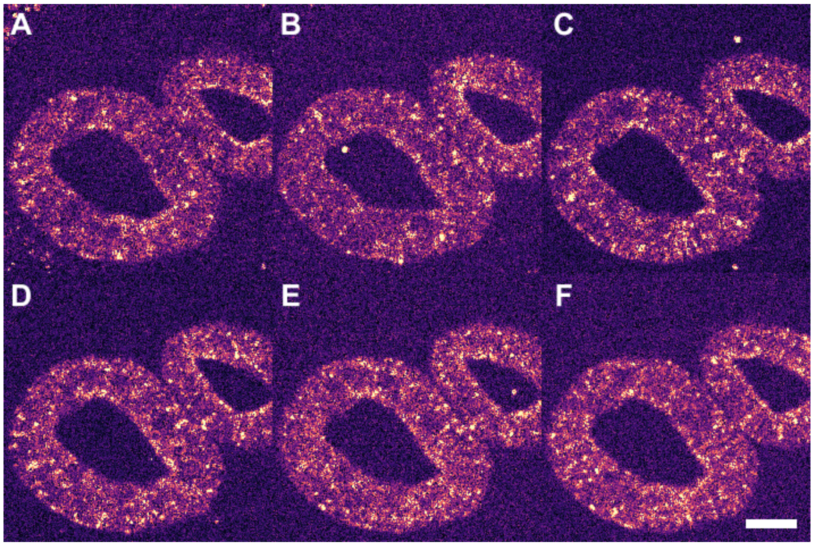

Fig. 1. Images reconstructed for different stage speeds with 200 raw frames acquired at 100 Hz. (A) v = 0.5 µm/s. (B) v = 1 µm/s. (C) v = 2 µm/s. (D) v = 3 µm/s. (E) v = 4 µm/s. (F) v = 5 µm/s. The CNR calculated for each image is presented in figure 2(B) of the article. Images were acquired using a 10X objective.

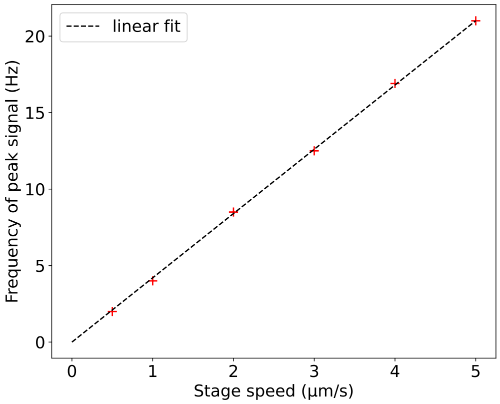

Fig. 2. Position of the maximum peak of the PSD as a function of the stage speed (red crosses) fitted with a linear function (black dashed line). We observe a linear relation with a coefficient $a = 4.2\mu m^{-1}$ while the theoretical value is $a = \frac{2n}{\lambda} = 4.25\mu m^{-1}$ for a source centered at 625nm and a refractive index of 1.33 (see subsection 3.1 for more information).

1.2. Same speed, different number of images per PSD

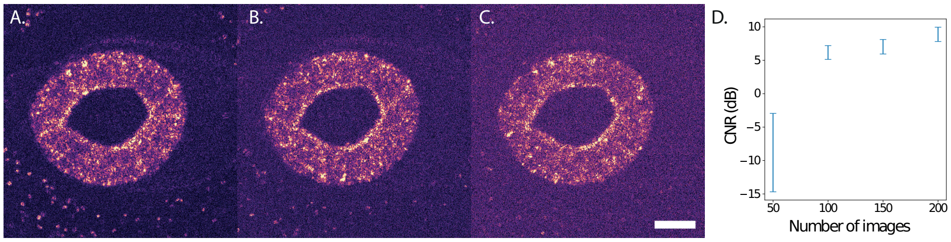

Fig. 3. Effect of the number of images on the CNR for a stage speed of 2 µm/s at a frame rat of 100 Hz. (A) Image reconstructed from 200 images, CNR = 9.8. (B) Image reconstructed from over 100 images, CNR = 6.6. (C) Image reconstructed from 50 images, CNR = 1.7. As expected, the CNR increases as we increase the number of images taken to calculate the PSD. Images were acquired using a 10X objective. (D) Average CNR measured for 10 successive planes as a function of the number of raw images used for reconstruction.

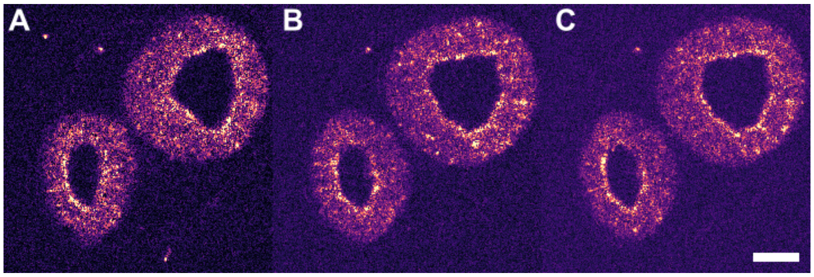

Fig. 4. Effect of considering sub-stacks to perform PSD measurement on the image quality. The experimental data presented here are the same as in the previous figure, except that we summed respectively 1, 2 and 4 successive calculated PSDs in order to reconstruct an image with the same number of raw frames. (A) PSD calculated over 200 images, CNR = 9.8. (B) PSD calculated over 100 images, with 2 successive reconstructed images summed together, CNR = 9.5. (C) PSD calculated over 50 images, with 4 successive reconstructed images summed together, CNR = 9.2. It appears that the best CNR is obtained by applying one single PSD over 200 frames rather than averaging over several PSD calculated on fewer frames. Images were acquired using a 10X objective.

1.3. Motor stability and its effect on the PSD

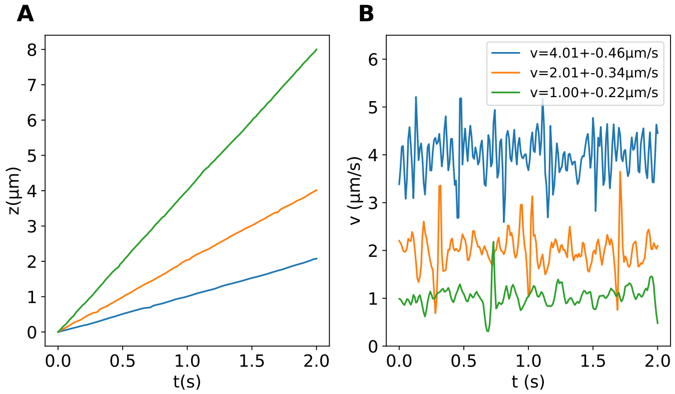

Fig. 5. Measurement of the actuator position and stability using an interferometer (Attocube, IDS3010). (A) Position and (B) speed as a function of time for speeds set at 1,2 and 4 µm/s.

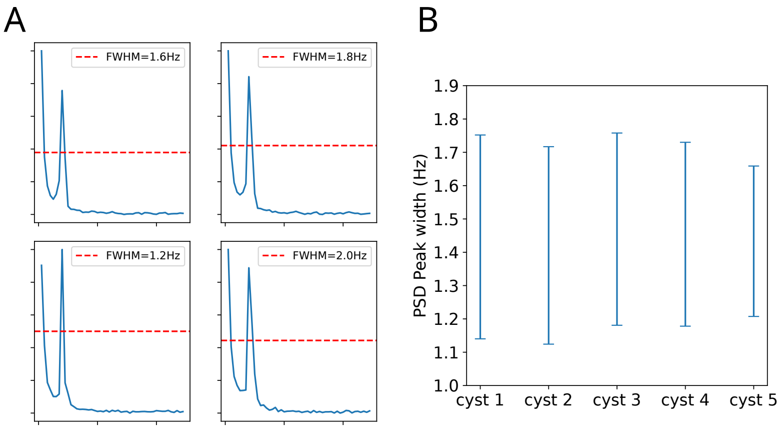

Fig. 6. Effect of motor stability on the PSD witdth. (A) Four average PSDs taken successively over 100 images with a motor speed of 2µm/s. We can see that the FWHM varies from one acquisition to another due to motor speed variations. (B) Average PSD width calculated for five different cysts in 40 successive planes imaged with identical conditions. We measure an average FWHM = 1.46 ± 0.28 Hz

1.4. Effect of reconstruction algorithm on continuous scanning FF-OCT images

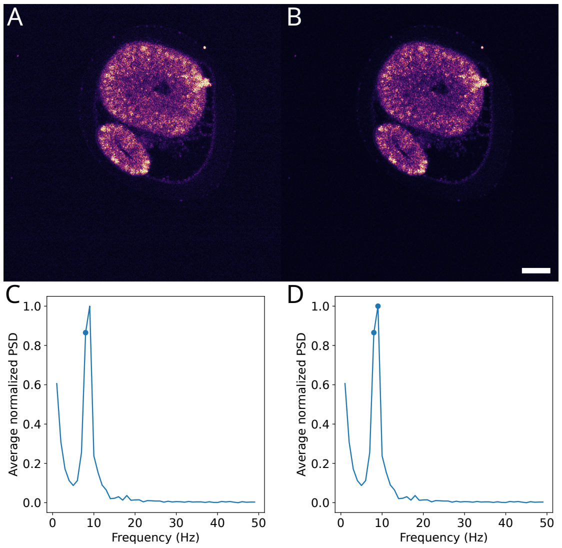

Fig. 7. Performances of the automatic peak detection algorithm. (A) and (B) display the same image plane of a fixed cyst reconstructed by taking respectively only the PSD value at the target frequency, represented in (C), and by taking the sum of the PSD values above a threshold, represented in (D) where two points of the PSD are considered. Taking into account several points in the PSD allows to compensate for motor speed instability leading to an increase in the PSD peak width. We increase the CNR by 3.8 dB, going from 13.8 dB to 17.6 dB. Images were acquired using a 20X objective.

2. Differences between four-phase FF-OCT and continuous scanning FF-OCT

2.1. Z-axis resolution and fringe artifacts of Continuous scanning FF-OCT vs four-phase FF-OCT

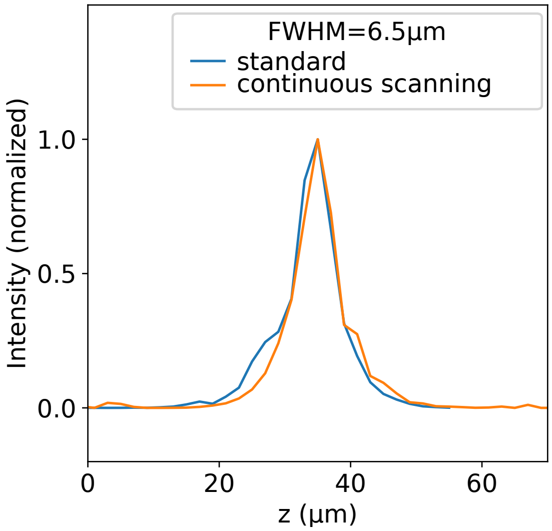

Fig. 8. Z-axis resolution for standard four-phase FF-OCT and continuous scanning FF-OCT. We observe an identical FWHM=6.5 µm for the two methods, showing that there is no loss in axial resolution with continuous scanning FF-OCT.

2.2. Contrast-to-noise ratio difference

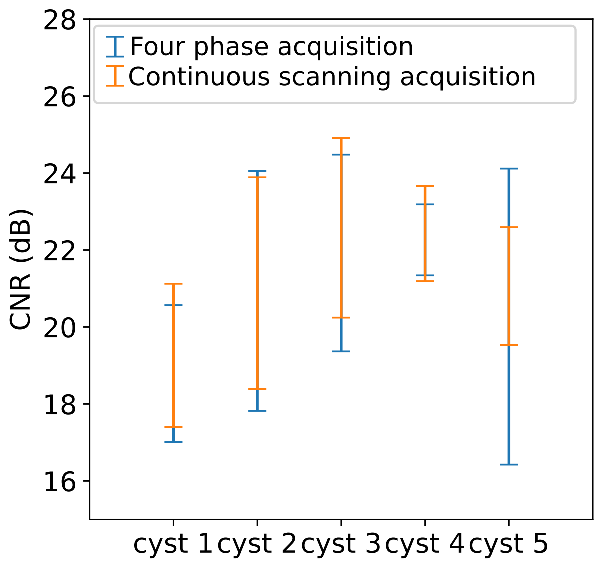

Fig. 9. Comparison of CNR between four-phase and continuous scanning FF-OCT images taken with respectively 96 images and 100 images, in 40 different planes for four different cysts. We observe an average gain of 0.6 dB for continuous scanning with a reduction of fringe artifacts. Images were acquired using a 20X objective.

3. Theory

3.1. Relationship between speed, signal and reflection coefficient

In OCT, the intensity on the detector at a given time t can be written as :

$$I = I_0 \left(R_r + R_{object} + 2\sqrt{R_r R_{object}} \cdot \cos\Phi \right) \tag{1}$$

where R_r is the reflection coefficient of the reference mirror and R_{object} is the reflection coefficient of the object in the interference plane, our signal of interest. The phase difference Φ is going to depend on the intrinsic phase Φ_0 of the object as well as on the time-dependent position of the motor:

$$\Phi = \Phi_0 + \omega_0 t = \Phi_0 + 2\pi\, f_0\, t = 2\pi \frac{2n\, v_0 t}{\lambda} \tag{2}$$

Here, the frequency f_0 is expressed as a function of the wavelength of the light source λ (625 nm), the speed of the stage v_0 and the refractive index n of the medium (typically 1.33 in water). If we acquire enough images over a distance greater than the wavelength and smaller than the coherence length of the light source with a sampling rate greater than two times the wavelength (which corresponds to a maximum speed of 5.9 µm/s for a framerate of 100 fps), we can use a pixel-by-pixel Fourier transform over time to retrieve the signal of interest in the time domain, i.e. calculate a power spectrum density (PSD):

$$\tilde{I}(f) = \frac{1}{\sqrt{2\pi}} \left(\int_{t_0}^{t_1} I(t)\, e^{-i\omega t}\, dt \right) \tag{3}$$

We then assume integration over a sufficiently large number of periods, allowing it to be treated as an integration over an infinite domain:

$$\tilde{I}(f) = \frac{I_0}{\sqrt{2\pi}} \left(\int_{-\infty}^{+\infty} 2\sqrt{R_r R_{object}}\, \cos(\Phi_0 + \omega_0 t)\, e^{i\omega t}\, dt \right) \tag{4}$$

If we only take into account positive frequencies, we can write :

$$\tilde{I}(f) = I_0 \sqrt{\frac{2 R_r R_{object}}{\pi}}\, \delta(\omega - \omega_0) \tag{5}$$

We thus expect to have a peak in the PSD when an object is present in the interference plane at the target frequency fixed by the stage speed. We use a light source with a coherence length of 10 µm. We can thus acquire images continuously over a distance shorter than the coherence length while staying in the interference plane. For example, at a speed $v_0 = 4$ µm/s, the expected target frequency would be 8.5 Hz.
